# Prevalence and distribution patterns of allergens among children with asthma and asthma-like symptoms in Shanghai, China

**DOI:** 10.1186/s12931-020-1318-1

**Published:** 2020-02-18

**Authors:** Song Mao, Liangxia Wu, Wenjing Shi

**Affiliations:** 1 Department of Pediatrics, Shanghai Jiao Tong University Affiliated Sixth People’s Hospital, 600 Yishan road, Shanghai, China; 2 China Hospital Development Institute, Shanghai Jiao Tong University, Shanghai, China

**Keywords:** Prevalence, Distribution, Allergens, Children, Asthma

## Abstract

**Objective:**

We aimed to identify the prevalence and distribution patterns of allergens among Chinese children with asthma/asthma-like symptoms (ALS).

**Methods:**

A total of 3479 children with asthma/ALS were enrolled. Skin prick test (SPT) was used to test the allergen-specific IgE. We analysed allergens prevalence and distribution, and its relationship with demographic characteristics.

**Results:**

Aeroallergens prevalence was higher than that of food allergens (*p* < 10^− 4^). Boys had higher aeroallergens prevalence than that in girls (p < 10^− 4^). Significant difference of aeroallergens prevalence among cases with different parental allergy history was observed (*p* < 10^− 4^). Age was positively associated with aeroallergens prevalence before the age of 11.5 (*P* < 10^− 4^), particularly before the age of 2.42 (P < 10^− 4^). Age was negatively associated with aeroallergens prevalence after the age of 11.5 (*P* = 0.021). Age was negatively associated with food allergens prevalence before the age of 3.42 (*P* < 10^− 4^). Age was associated with the intensity of dermatophagoides farinae (DF)/house dust mite (HDM) allergens (*P* < 10^− 4^). Age was negatively associated with the intensity of shrimp, and crab allergens before the age of 3.3 and 3.3, respectively (*P* = 0.012, < 10^− 4^). Boys had higher intensity of DF and HDM allergens than that in girls (*P* < 10^− 4^, P < 10^− 4^). Significant differences of the intensity of DF and HDM allergens among groups with different parental allergy history were noted (*P* < 10^− 4^, P < 10^− 4^).

**Conclusions:**

Boys and parental allergy history were associated with higher prevalence and intenstity of aeroallergens. Age was positively and negatively associated with aeroallergens prevalence before and after the age of 11.5, respectively. Age was negatively associated with food allergens prevalence before the age of 3.42.

## Introduction

Asthma, an allergic, immunogenic and inflammatory disease, occurs commonly in children [1]. Asthma prevalence has increased across the world due to multiple factors, including environmental changes, increased outdoor activities and climate changes [2]. It is likely to lead to great morbidity, even affecting the growth and development of children. Meanwhile, many children presented with atypical symptoms, such as intermittent or persistent wheezing, called asthma-like symptoms (ALS), prone to develop asthma [3]. Due to the potential harms of asthma/ALS, an in-depth investigation of the risk factors for asthma/ALS susceptibility was of great implications.

Allergic response, direct result of reaction induced by a sensitized host immune system to a specific allergen, plays an important role in the pathogenesis of asthma/ ALS [4]. The allergens activate the immune response by interacting with effector immune cells and promoting a Th2-skewed activation of T cells, leading to the IgE-mediated sensitization [5]. In this sense, allergens are closely associated with the susceptibility to asthma/ALS, allergens avoidance is vital for asthma/ALS prevention. Hence, identification of the allergens is helpful for the management of asthma/ALS.

In the past decades, lots of studies have been focused on common allergens testing in the specific populations [6–8]. There are different allergen sensitizaton patterns in various regions. People can benefit from allergens testing because immunotherapy depends on the testing results of allergens. Meanwhile, identification of the risk factors for prevalent allergens is of great guidance for high-risk populations. Previous studies regarding the allergens testing focused mainly on the adults, we decided to perform a cross-sectional study regarding the prevalence and distribution patterns of allergens in Chinese children with asthma/ALS.

Skin prick test (SPT) of allergens can reflect the allergic test results directly and objectively in the skin. We can also test the response diameter of specific allergen in the skin, which reflect the intensity of allergic reaction. Hence, we used the SPT method to identify the prevalent allergens in the children with asthma/ALS. We also analyzed the chronological trend of prevalent allergens, the impact of gender, age and parental allergy history on the distribution patterns of prevalent allergens.

## Methods

### Patient population

The study population aged 0–16 years diagnosed with asthma or ALS in our center between January 2010 and December 2018 were screened. Asthma was defined as a clinical syndrome consisting of wheeze, breathlessness, chest tightness and sometimes cough [9]. ALS was defined as acute attack of cough, wheezing or short of breath within one year prior to enrollment in this study. Parental allergy history was defined as having at least one of the followings: asthma, allergic rhinitis, allergic eye symptoms or atopic dermatitis. A total of 3479 cases received the SPT tests. The data of enrolled subjects was de-identified. This study was approved by the institutional review board. The informed consent of guardians of subjects was obtained. This cross-sectional study was performed in terms of the Declaration of Helsinki.

### Data collection

We extracted the clinical data of all the enrolled subjects from the medical records.

Clinical and demographic data were reviewed separately for age and gender. The data included the study year, age, gender, and parental allergy history. The data was obtained before the SPT of allergens. Age were divided into 0–3, 3–6, 6–9, 9–12, and 12- groups. Based on the parental allergy history, the cases were divided into four groups of none (neither father nor mother had allergy history), mother only (only mother had allergy history), father only (only father had allergy history), and F-M (both father and mother had allergy history). Allergens types were divided into aeroallergens and food allergens. Testing results of specific allergens were expressed as negative or positive, as well as the the length of diameter for the allergic response for antigen of specific allergen. The histamine was used as the positive control with physiological saline as the negative control.

### Statistical analysis

Continuous variables were expressed as the mean and standard deviation (SD) in the normal distribution or median/quartile in the non-normal distribution. Categorical variables were expressed as the frequency. Student’s t tests and one-way anova tests were applied to investigate the differences of continuous variables among different groups. Chi-square test was used to investigate the differences of categorical variables among different groups. We performed piecewise linear regression models to examine the threshold effects of age on the positive rate of aeroallergens/food allergens, and the allergen response diameter according to the smoothing plot with the adjustment for the gender and parental allergy history. The threshold level was determined using a trial method. The infection point that gave the maximum modle likelihood was detected. Sensitivity analysis was performed by detecting the trend of the association between parental allergy history and aeroallergens/food allergens positive rate. The statistical analyses were conducted using the software R and EmpowerStats (http:// www. empowerstats.com, X&Y Solutions, Inc., Boston, MA). The value of p less than 5% was considered statistically significant.

## Results

### Baseline characteristics and allergens distribution of participants

**Fig. 1:**
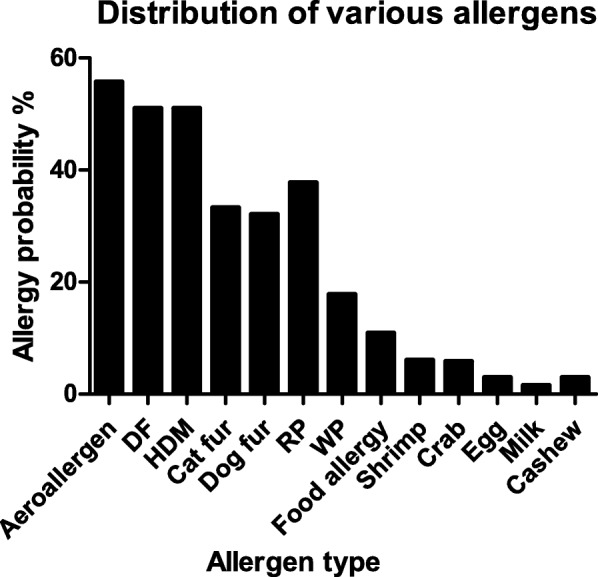
Distribution of the probability of various allergens in children with asthma/ALS

A total of 3479 asthma/ALS cases including 2022 boys and 1457 girls were enrolled. The median age was 4.50 (3.50, 6.00). 1177 cases presented without parental allergy history, 1074 cases presented with only father allergy history, 879 cases presented with only mother allergy history, and 349 cases presented with allergy history of both father and mother. The most common aeroallergens for asthma/ALS were dermatophagoides farinae (DF) and house dust mite (HDM), the most common food allergens were shrimp and crab. The positive rate of aeroallergens was significantly higher than that of food allergens (*p* < 10^− 4^). The chronological changes of allergens distribution in asthma/ALS were presented in Table 1 and Fig. 1.

**Table 1:** Baseline characteristics of recruited participants in various years

Year	N	Boy/girl	Age	Father	Mother	F-M	None					
**2010**	**467**	**281/186**	**4.00 (3.00,5.50)**	**128**	**109**	**22**	**208**					
**2011**	**341**	**200/141**	**4.00 (3.00,5.50)**	**105**	**73**	**33**	**130**					
**2012**	**293**	**169/124**	**4.50 (3.50,6.50)**	**91**	**80**	**29**	**93**					
**2013**	**178**	**103/75**	**4.50 (3.63,6.00)**	**64**	**35**	**17**	**62**					
**2014**	**331**	**209/122**	**4.50 (3.50,6.00)**	**110**	**95**	**29**	**97**					
**2015**	**279**	**163/116**	**4.50 (3.50,6.50)**	**107**	**66**	**20**	**86**					
**2016**	**432**	**260/172**	**5.00 (4.00,6.50)**	**122**	**128**	**56**	**126**					
**2017**	**551**	**298/253**	**5.00 (3.50,6.25)**	**170**	**129**	**57**	**195**					
**2018**	**607**	**339/268**	**5.00 (4.00,7.00)**	**177**	**164**	**86**	**180**					
**Total**	**3479**	**2022/1457**	**4.50 (3.50,6.00)**	**1074**	**879**	**349**	**1177**					
**Year (N)**	**Total (N)**	**DF (N)**	**HDM (N)**	**Cat fur (N)**	**Dog fur (N)**	**RP (N)**	**WP (N)**	**Shrimp (N)**	**Crab (N)**	**Egg (N)**	**Milk (N)**	**Cashew (N)**
**2010**	**467**	**250**	**241**	**168**	**163**	**86**	**57**	**63**	**46**	**10**	**5**	**28**
**2011**	**341**	**176**	**176**	**143**	**128**	**80**	**40**	**21**	**20**	**8**	**3**	**10**
**2012**	**293**	**163**	**162**	**116**	**112**	**111**	**56**	**17**	**14**	**7**	**4**	**17**
**2013**	**178**	**87**	**89**	**67**	**64**	**76**	**42**	**7**	**8**	**5**	**4**	**5**
**2014**	**331**	**190**	**194**	**138**	**129**	**169**	**67**	**39**	**41**	**12**	**6**	**7**
**2015**	**279**	**127**	**129**	**86**	**87**	**123**	**61**	**18**	**18**	**13**	**6**	**5**
**2016**	**432**	**215**	**223**	**139**	**137**	**189**	**85**	**15**	**20**	**14**	**3**	**7**
**2017**	**551**	**257**	**255**	**125**	**125**	**217**	**94**	**13**	**21**	**21**	**10**	**11**
**2018**	**607**	**309**	**307**	**176**	**172**	**261**	**118**	**17**	**13**	**12**	**11**	**11**
**Total**	**3479**	**1774**	**1776**	**1158**	**1117**	**1312**	**620**	**210**	**201**	**102**	**52**	**101**

Age: median (Q1, Q3), Family allergy history (Father: father allergy only, Mother: mother allergy only, F-M: both of father and mother allergy, none: neither of father nor mother allergy)

*DF* dermatophagoides farinae, *HDM* house dust mite, *RP* ragweed, *WP* willow

### Association between demographic characteristics and allergens distribution

**Fig. 2:**
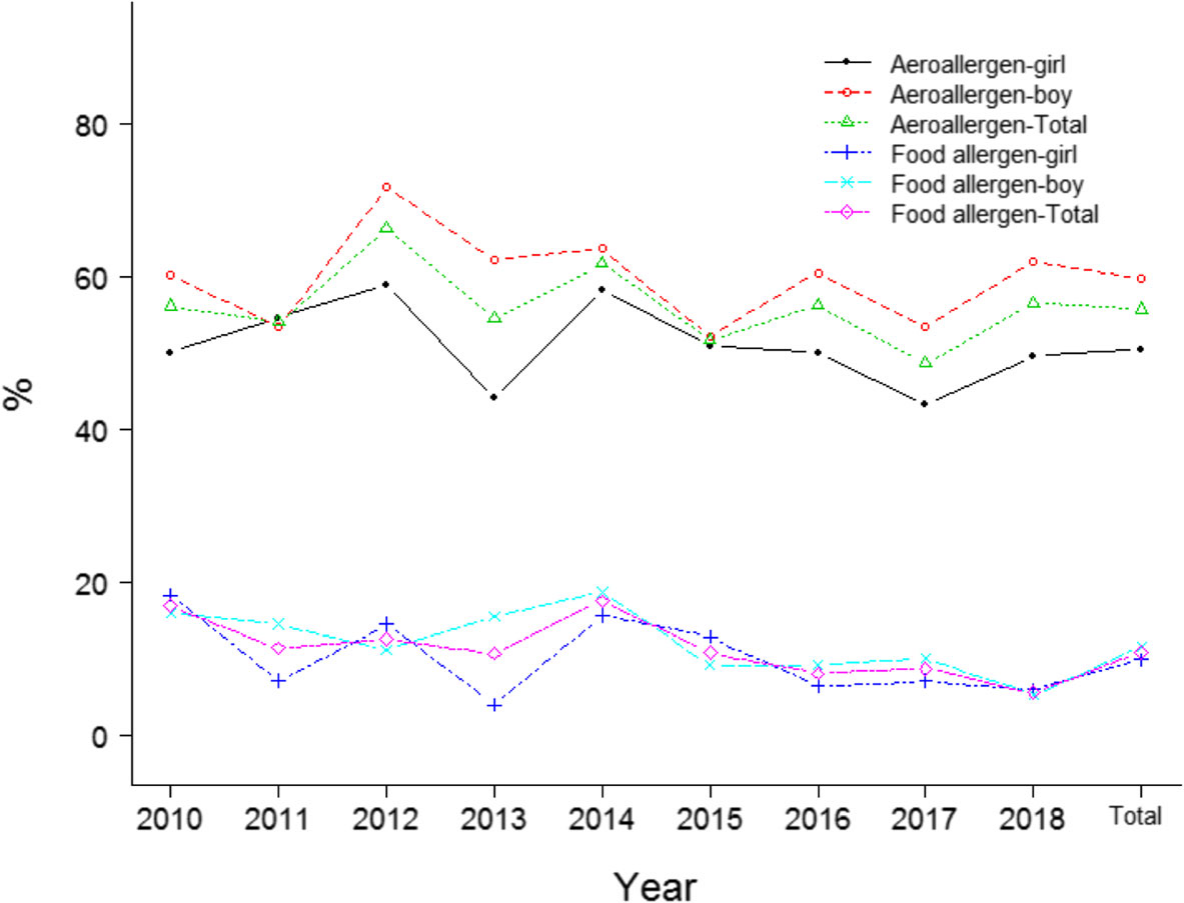
Distribution the probability of aeroallergen/food allergen in children with asthma/ALS

**Fig. 3:**
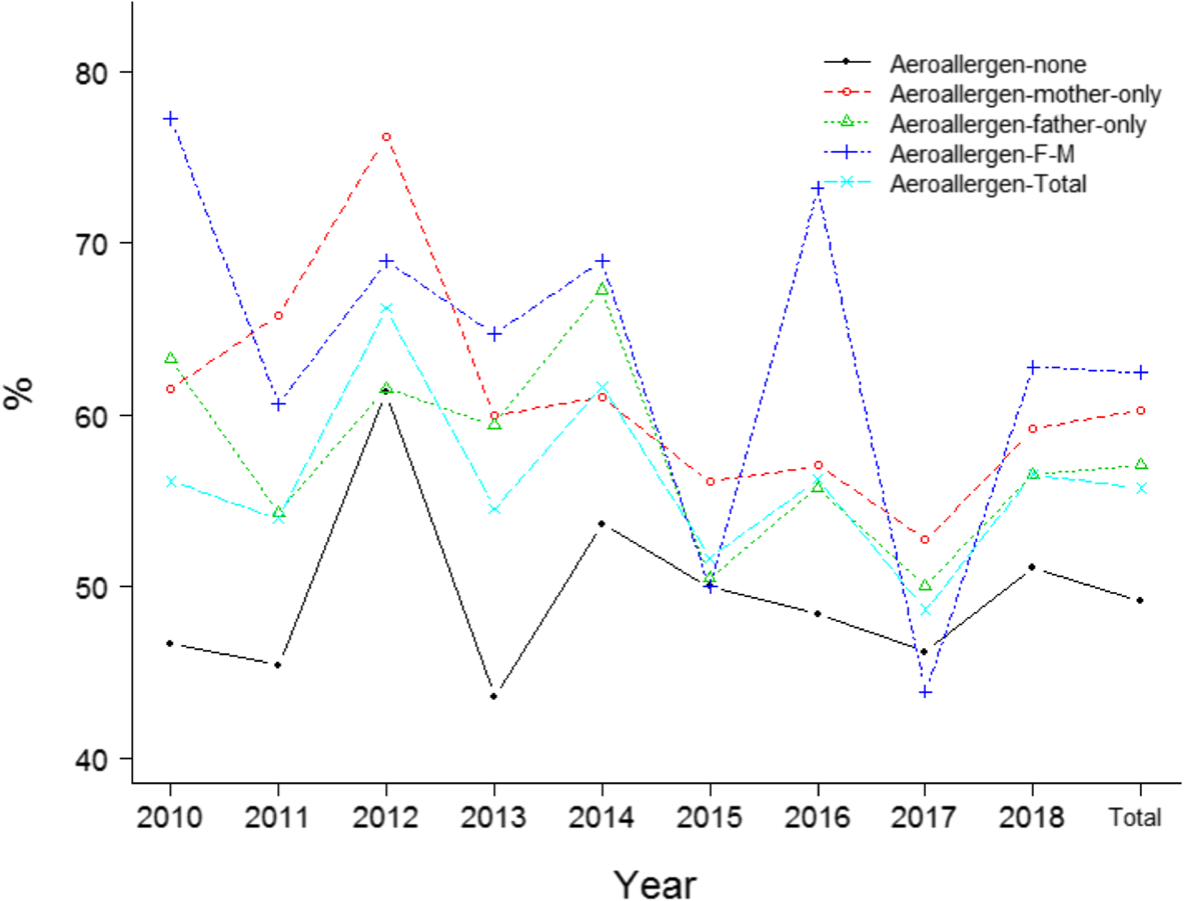
Distribution the probability of aeroallergen in children with asthma/ALS

**Fig. 4:**
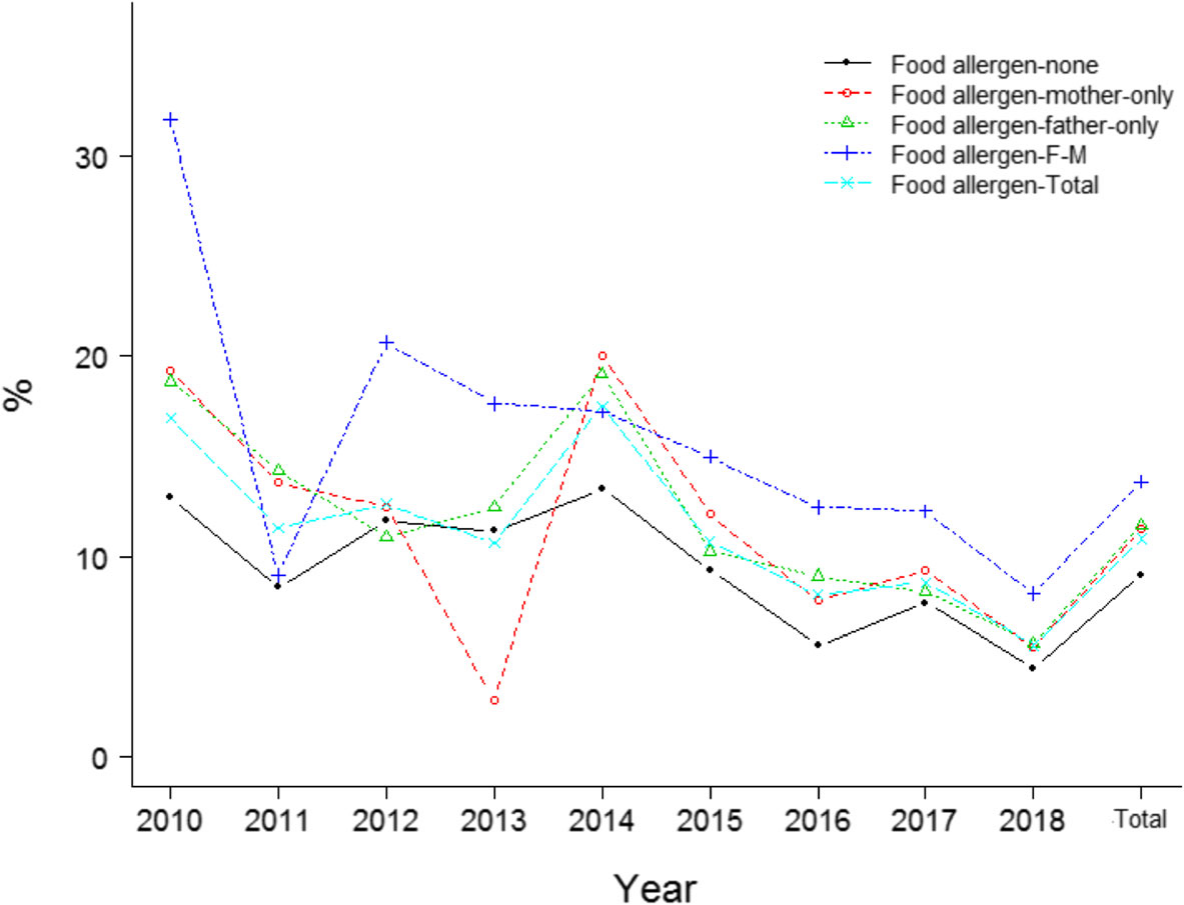
Distribution the probability of food allergen in children with asthma/ALS

**Fig. 5:**
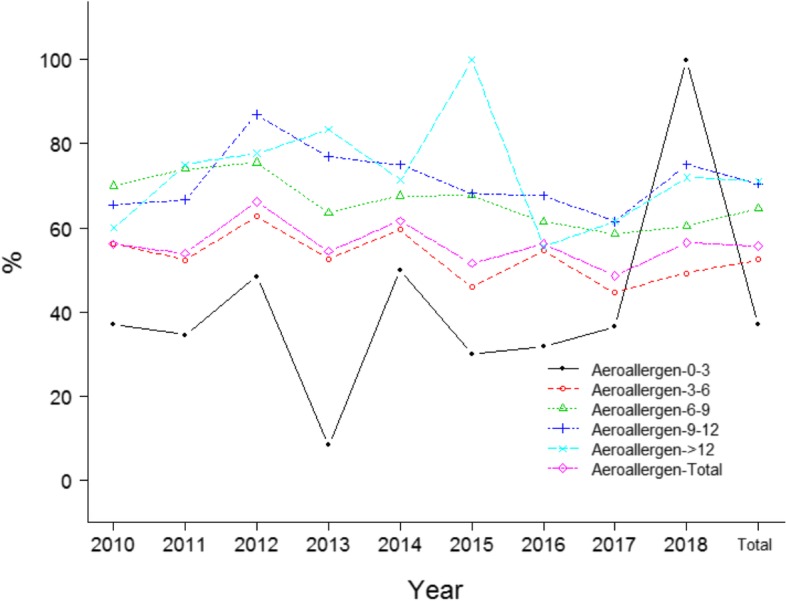
Distribution the probability of aeroallergen in children with asthma/ALS

The positive rate of aeroallergens in boys was significantly higher than that in girls among the total population (*p* < 10^− 4^, Fig. 2), a similar trend was seen in various years (Fig. 2). No significant difference of the positive rate of food allergens between boys and girls was observed among the total population (*p* = 0.104, Fig. 2). Significant difference of the positive rate of aeroallergens among cases with different parental allergy history was noted in the total population (*p* < 10^− 4^, Fig. 3). No marked difference of the positive rate of food allergens among cases with different parental allergy history was observed in the total population (*p* = 0.056, Fig. 4). Significant difference of the positive rate of aeroallergens among cases with different age groups was noted in the total population (*p* < 10^− 4^, Fig. 5). No marked difference of the positive rate of food allergens among cases with different age groups was noted in the total population (*p* = 0.056, Supplemental material (SM) 1).

### Non-linear relationship between age and positive rate of aeroallergens/food allergens

Age was positively associated with the positive rate of aeroallergens before the infection point of 11.5 years of age (*P* < 10^− 4^), particularly before the infection point of 2.42 years of age (*P* < 10^− 4^). Age was negatively associated with the positive rate of aeroallergens after the infection point of 11.5 years of age (*P* = 0.021). Age was significantly associated with the positive rate of aeroallergens after adjusting for gender and parental allergy history (*P* < 10^− 4^, SM 2). Age was negatively associated with the positive rate of food allergens before the infection point of 3.42 years of age (*P* < 10^− 4^, SM 3), age was not associated with the positive rate of food allergens after the infection point of 3.42 years of age (*P* = 0.128).

### Relationship between demographic characteristics and allergens intensity

Age was significantly associated with the intensity of DF and HDM allergen (*P* < 10^− 4^, SM 4–5). Age was negatively associated with the intensity of shrimp, and crab allergen before the infection point of 3.3 and 3.3, respectively (*P* = 0.012, and *P* < 10^− 4^, SM 6–7). Boys had significantly higher intensity of DF and HDM allergen than that in girls (*P* < 10^− 4^, and *P* < 10^− 4^). There was no marked differences of shrimp and crab allergens between boys and girls (*P* = 0.519, and *P* = 0.649). Significant differences of the intensity of DF and HDM allergens among groups with different parental allergy history were noted (*P* < 10^− 4^, and *P* < 10^− 4^, SM 8–9). Three dimentional bar chart showed that boys with F-M allergy had the highest intensity of DF and HDM allergen. Notably, no marked differences of the intensity of DF and HDM allergens between father-only and moher-only group (*P* = 0.99, and *P* = 0.99, SM 10–11). There was no marked differences of shrimp and crab allergens among groups with different parental allergy history (*P* = 0.716, and *P* = 0.35).

## Discusssion

Asthma usually arises in early childhood. Asthma prevalence has been increasing in the past decades. Meanwhile, many cases presented with ALS, a symptom likely to leading to the occurrence of asthma. Increased IgE status is often observed in asthma/ALS. The exaggerated response of host immune systems to foreign antigens is a main cause of asthma/ALS. Allergens exposure may lead to the excessive IgE production and cellular responses, a trigger of asthma/ALS attack [10]. Hence, identification and avoidance of the prevalent allergens is helpful for the prevention and treatment of asthma/ALS.

Our study showed that aeroallergens were the most prevalent allergens in asthma/ALS, age was closely associated with the prevalence/intensity of aeroallergens, a turning point of age existed between its relationship with aeroallergens, and boys/parental allergy history were associated with higher prevalence/intensity of aeroallergens. Our findings were of great implications that the demographic characteristics may be associated with allergens prevalence and intensity, asthma/ALS cases with different demographic characteristics showed different allergens distribution, and it may be useful for the prediction of prevalent allergens in asthma/ALS.

Several facts may account for our findings. First, asthma is a pulmonary disease characterized by chronic airway inflammation and hyperresponsiveness, inhalant allergens can directly trigger the respiratory allergy symptoms [11]. Sensitization to aeroallergens is likely to lead to the decreased lung function and nasal patency [12], indicating aeroallergens may be more likely to increase the susceptibility to asthma/ ALS, which was consistent with our findings that aeroallergens were more prevalent in asthma/ALS. Food allergy was not common in children with asthma/ALS due to the facts that food allergens did not affect the respiratory tract system directly. We also found that the positive rate of food allergens was higher in the infantile period than that in other periods, which may be due to that the digestive enzymes were not mature and the infants needs to adapt to various food patterns in this period [13]. On the other hand, the most common aeroallergens were DF and HDM, which was consistent with the epidemiological findings that mites were the most common allergens in China [14]. Second, boys were more sensitive to aeroallergens than girls regardless of the years, which may be due to that boys were likely to have more activities than girls, resulting in more aeroallergens exposure. No significant differences of positive rate of food allergens were observed between boys and girls, which may be due to that there was no marked differences of food patterns between boys and girls. Parental allergy history affected the positive rates of both aeroallergens and food allergens in children with asthma/ALS, which was consistent with the idea that hereditary factors influenced the allergy status [15], affecting the allergens distribution. Notably, we also found that parental allergy history and gender were closely associated with intensity of DF/HDM allergens, F-M and boys group showed the highest intensity, which indicated that more attention should be paid to this group in the prevention of asthma/ALS. Finally, we observed that age was negatively associated with the positive rate of food allergens before the infection point of 3.42 years of age, whereas age was not associated with the positive rate of food allergens after the infection point of 3.42 years of age, which may be explained by the facts that food patterns changes a lot during the infantile period, leading to a higher positive rate of food allergens before this period [16]. Meanwhile the food patterns were comparatively stable after infantile period. For aeroallergens, the group aged 0–3 years showed the lowest positive rate of aeroallergens in various years except 2018, which may be due to the small number of participants in 2018. Interestingly, we found that age was positively associated with the positive rate of aeroallergens before the infection point of 11.5 years, particularly before the infection point of 2.42 years, whereas age was negatively associated with the positive rate of aeroallergens after the infection point of 11.5 years. This finding may be due to the facts that the scope of activities was increasing with the growing of children, particularly in the infantile period, leading to more exposure to aeroallergens, meanwhile the immunity is increasing with the growing of the children, reaching a stable status during adolescence, leading to a decline of positive rate of aeroallergens.

Our findings were of obvious strengths that we can made an initial evaluation of the allergens patterns and distribution in the specific population, and early avoidance and intervention was feasible, particularly for the population without allergens test due to various reasons. Meanwhile, we also can pay more attention to the high-risk population, such as boys and F-M group. However, several issues arised. First, the specific role of allergens in the asthma/ALS needs an in-depth study. In terms of the prevalence and distribution patterns of allergens in asthma/ALS, we speculated that aeroallergens, particularly DF and HDM were the most common triggers of asthma/ ALS; age, gender and parental allergy history affected the allergens prevalence and distribution. Hence, we speculated the the factors of growth and development, and immunity status played a role in the asthma/ALS attack through affecting the allergens tolerance. Second, we found that the positive rate of aeroallergens declined after a turning point of age. We speculated that the allergens tolerance reached a stable state after the turning point, and the positive rate of aeroallergens may decline naturally after this age point. Therefore, we speculated that the immunotherapy for specific allergen may be performed in an appropriate age. Finally, allergens may influence the inflammatory and immunity status, and the interaction between allergens and other factors may further influence asthma/ALS risk. The detailed role of allergens in asthma/ALS merits further investigation.

In the past decades, lots of studies were conducted to investigate the allergens sensitization patterns. Luo et al. [17] reported that house dust mite was the most common allergen in southern China, which was consistent with our findings. Lee [18] reported that different aeroallergens had different effects on the upper and lower airways of inner-city children in Korea, aeroallergen sensitization was closely related to clinical presentation. Di et al. [19] reported that significant differences were observed between the children and adults. We also found a turning point of age regarding its association with aeroallergens prevalence. It further proved that age had a impact on the positive rate of aeroallergens. Ibrahim et al. [20] reported that family history and female gender were the predictors of allergy, which was contrast to our findings that boys had significantly higher positive rate of aeroallergens than that in girls. The participants enrolled in the study of Ibrahim et al. were mainly adults. The differences between adults and children may explain our findings. Several limitations merit our consideration. The comparatively small number of participants limited the statistical power. The study design was cross-sectional analysis, the recall bias may be inevitable. Future studies may be needed to address these issues.

## Conclusion

Our study indicated that aeroallergens, particularly DF and HDM, were highly prevalent in children with asthma/ALS. Boys and parental allergy history were associated with higher prevalence and intenstity of aeroallergens. Age was positively associated with the prevalence of aeroallergens before the turning point of age of 11.5, and negatively associated with the prevalence of aeroallergens after this turning point. Age was negatively associated with the positive rate of food allergens before the infection point of 3.42 years of age. Our findings were of great implications that demographic characteristics were closely associated with the allergens distribution, prevalence and intensity, and these characteristics were helpful for predicting the allergic status in asthma/ALS.

## Supplementary information

**Additional file 1.** Supplemental material 1: Distribution the probability of food allergen in children with asthma/ALS. Supplemental material 2: Non-linear relationship between age and the probability of aeroallergen. Supplemental material 3: Non-linear relationship between age and the probability of food allergen. Supplemental material 4: Non-linear relationship between age and the intensity of DF allergen. Supplemental material 5: Non-linear relationship between age and the intensity of HDM allergen. Supplemental material 6: Non-linear relationship between age and the intensity of shrimp allergen. Supplemental material 8: The intensity of DF allergen among groups with different parental allergy history. Supplemental material 9: The intensity of HDM allergen among groups with different parental allergy history. Supplemental material 10: The intensity of DF allergen among different groups. Supplemental material 11: The intensity of HDM allergen among different groups.

## Data Availability

The data will be shared upon the scientifc request.

## Abbreviations

ALS: Asthma like symptoms
DF: Dermatophagoides farinae
HDM: House dust mite
SD: Standard deviation
SM: Supplemental material
SPT: Skin prick test
