# Supplementary material for: Prevalence and distribution patterns of allergens among children with asthma and asthma-like symptoms in Shanghai, China
Source: Respir Res. 2020 Feb 18;21:57. doi: 10.1186/s12931-020-1318-1 (PMC7029476; doi:10.1186/s12931-020-1318-1)
Supplement: Supplementary file 1 — Additional file 1. Supplemental material 1: Distribution the probability of food allergen in children with asthma/ALS. Supplemental material 2: Non-linear relationship between age and the probability of aeroallergen. Supplemental material 3: Non-linear relationship between age and the probability of food allergen. Supplemental material 4: Non-linear relationship between age and the intensity of DF allergen. Supplemental material 5: Non-linear relationship between age and the intensity of HDM allergen. Supplemental material 6: Non-linear relationship between age and the intensity of shrimp allergen. Supplemental material 8: The intensity of DF allergen among groups with different parental allergy history. Supplemental material 9: The intensity of HDM allergen among groups with different parental allergy history. Supplemental material 10: The intensity of DF allergen among different groups. Supplemental material 11: The intensity of HDM allergen among different groups. [file 12931_2020_1318_MOESM1_ESM.doc]

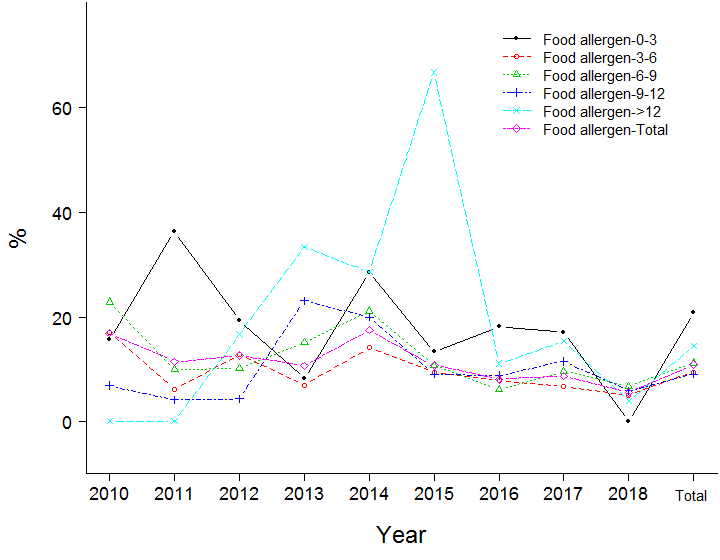


Supplemental material 1: Distribution the probability of food allergen in children with asthma/ALS


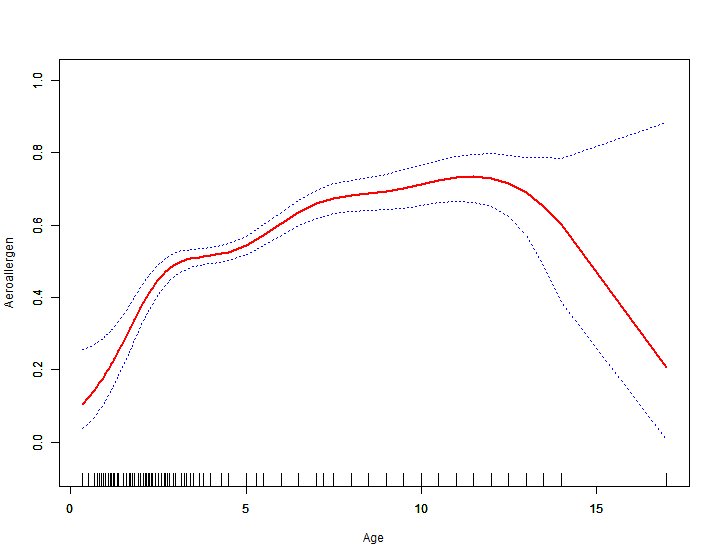


Supplemental material 2: Non-linear relationship between age and the probability of aeroallergen


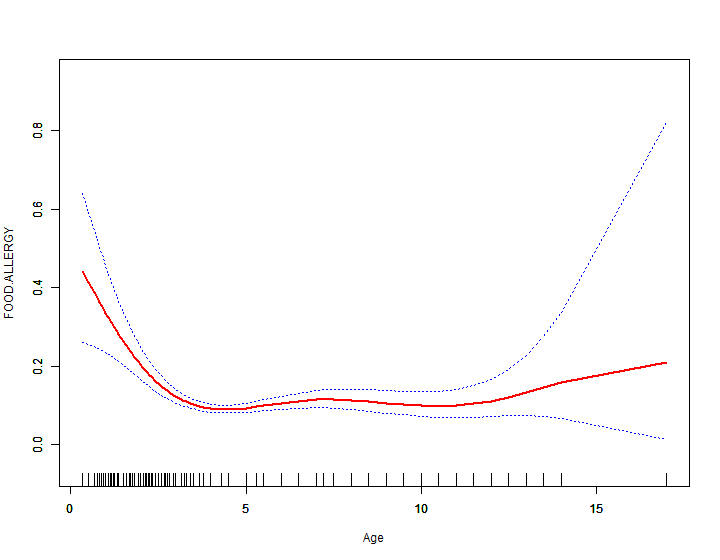


Supplemental material 3: Non-linear relationship between age and the probability of food allergen


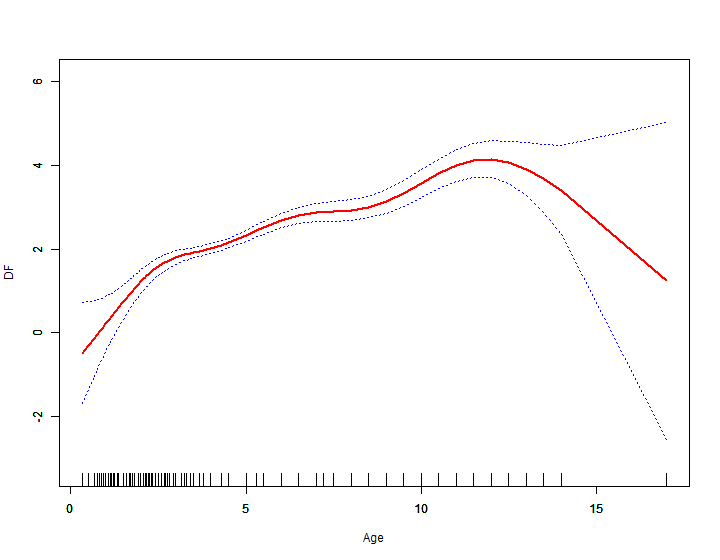


Supplemental material 4: Non-linear relationship between age and the intensity of DF allergen


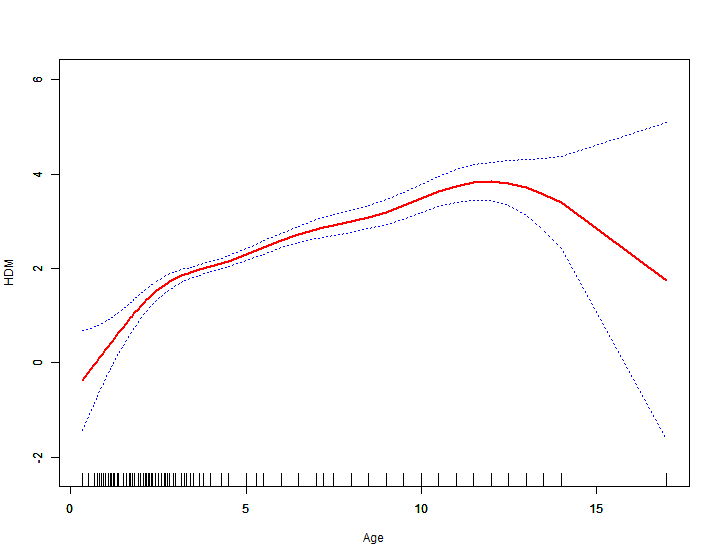


Supplemental material 5: Non-linear relationship between age and the intensity of HDM allergen


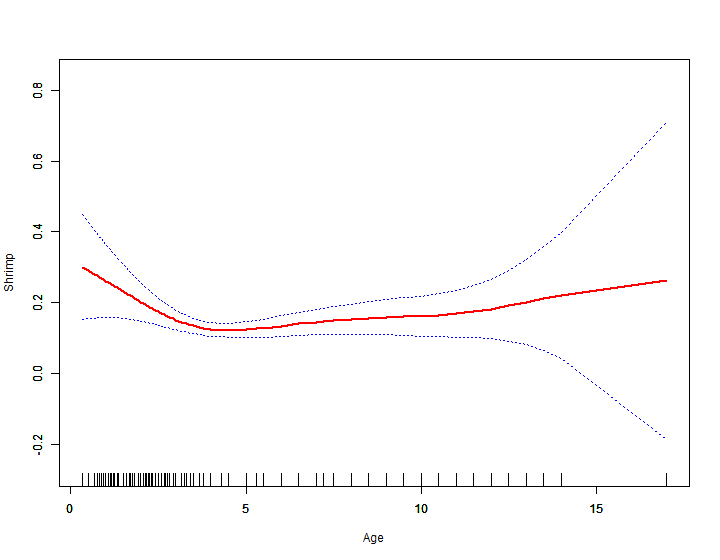


Supplemental material 6: Non-linear relationship between age and the intensity of shrimp allergen


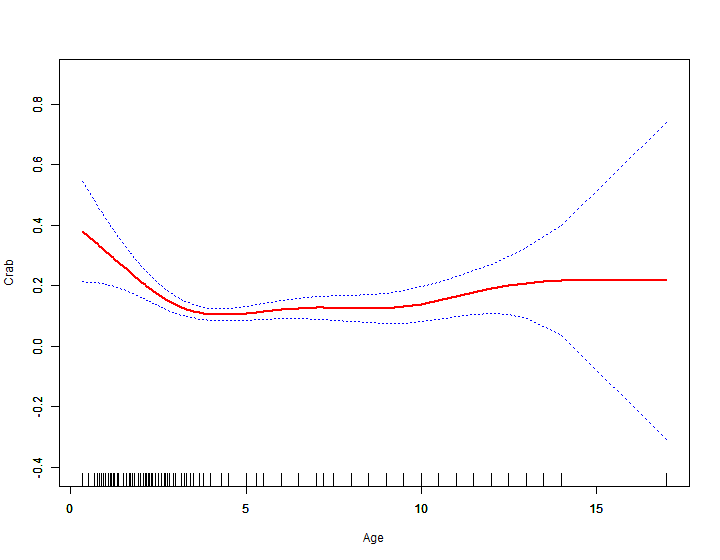


Supplemental material 7: Non-linear relationship between age and the intensity of crab allergen


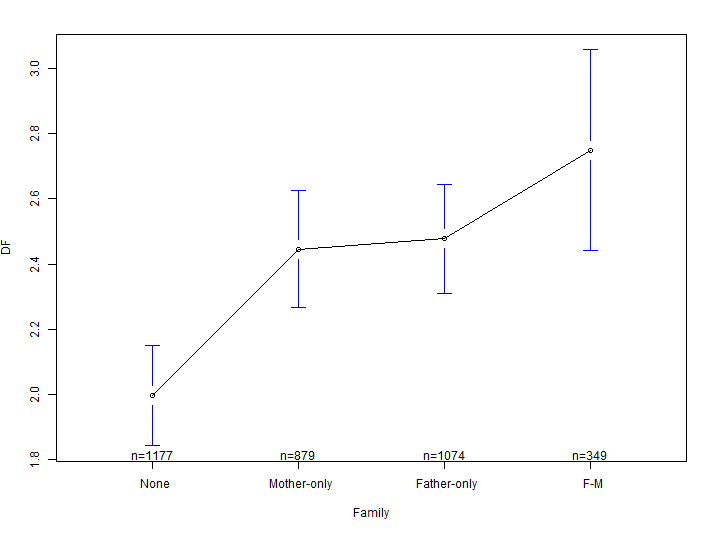


Supplemental material 8: The intensity of DF allergen among groups with different parental allergy history


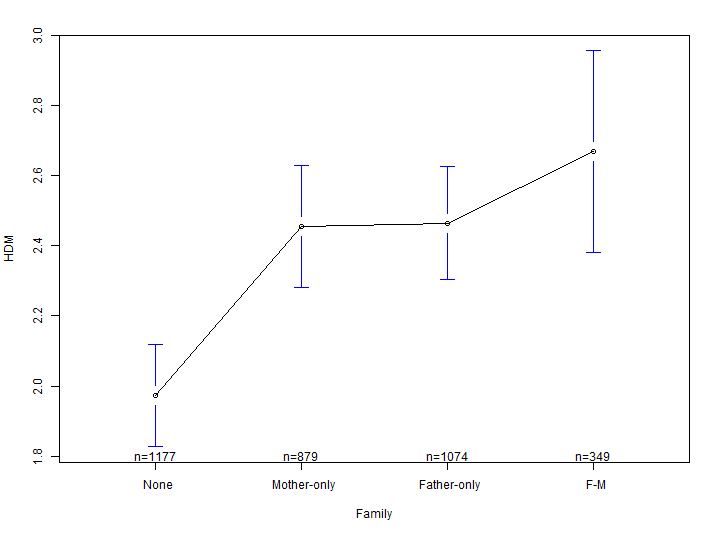


Supplemental material 9: The intensity of HDM allergen among groups with different parental allergy history


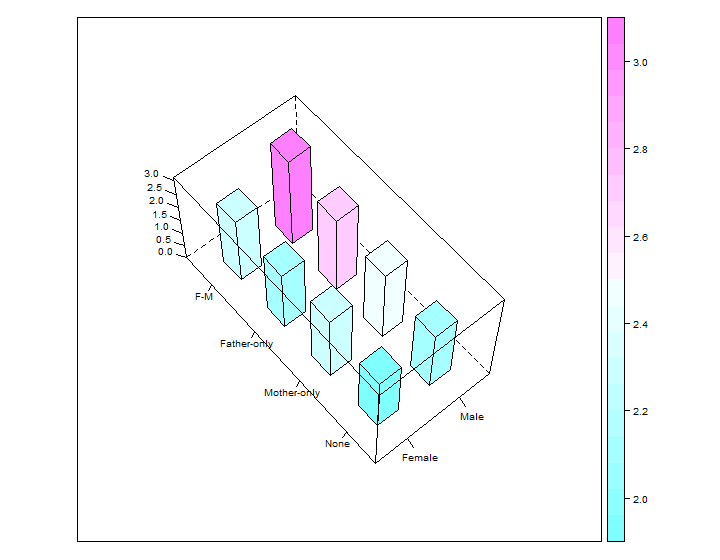


Supplemental material 10: The intensity of DF allergen among different groups


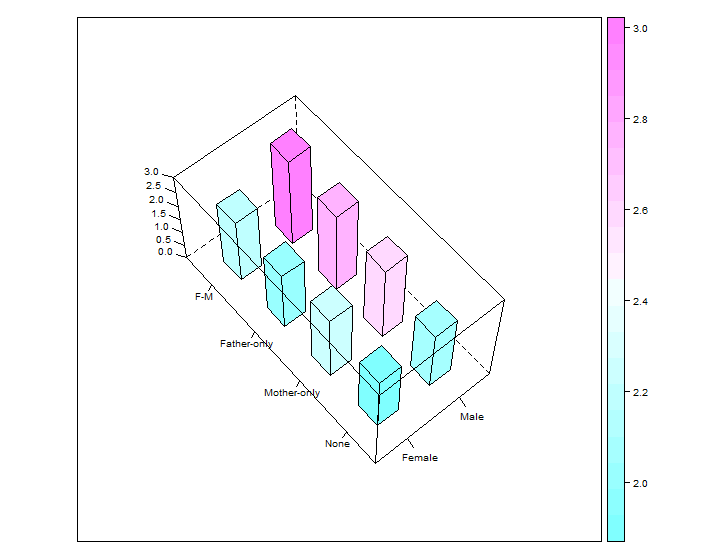


Supplemental material 11: The intensity of HDM allergen among different groups
